# High BCAR1 expression is associated with early PSA recurrence in ERG negative prostate cancer

**DOI:** 10.1186/s12885-017-3956-3

**Published:** 2018-01-05

**Authors:** Asmus Heumann, Nina Heinemann, Claudia Hube-Magg, Dagmar S. Lang, Katharina Grupp, Martina Kluth, Sarah Minner, Christina Möller-Koop, Markus Graefen, Hans Heinzer, Maria Christina Tsourlakis, Waldemar Wilczak, Corinna Wittmer, Frank Jacobsen, Hartwig Huland, Ronald Simon, Thorsten Schlomm, Guido Sauter, Stefan Steurer, Patrick Lebok, Andrea Hinsch

**Affiliations:** 1 Institute of Pathology, University Medical Center Hamburg-Eppendorf, Martinistr. 52, 20246 Hamburg, Germany; 2 General, Visceral and Thoracic Surgery Department and Clinic, University Medical Center Hamburg-Eppendorf, Martinistr. 52, Hamburg, Germany; 3 Martini-Clinic, Prostate Cancer Center, University Medical Center Hamburg-Eppendorf, Martinistr. 52, Hamburg, Germany; 4 Department of Urology, Section for translational Prostate Cancer Research, University Medical Center Hamburg-Eppendorf, Martinistr. 52, Hamburg, Germany

**Keywords:** BCAR1, Prostate cancer, Tissue microarray, Prognosis, Immunohistochemistry

## Abstract

**Background:**

Breast cancer anti-estrogen resistance 1 (BCAR1/p130cas) is a hub for diverse oncogenic signaling cascades and promotes tumor development and progression.

**Methods:**

To understand the effect of BCAR1 in prostate cancer, we analyzed its expression on more than 11,000 prostate cancer samples. BCAR1 expression levels were compared with clinical characteristics, PSA recurrence, molecular subtype defined by ERG status and 3p, 5q, 6q and PTEN deletion.

**Results:**

BCAR1 staining was barely detectable in normal prostate glands but seen in 77.6% of 9472 interpretable cancers, including strong expression in 38.5%, moderate in 23.2% and weak in 15.9% of cases. BCAR1 up regulation was associated with positive ERG status (*p* < 0.0001), high Gleason score (*p* < 0.0001), advanced pathological tumor stage (*p* = 0.0082), lower preoperative PSA level (*p* < 0.0001), increased cell proliferation (*p* < 0.0001), early PSA recurrence (*p* = 0.0008), and predicted prognosis independently from clinico-pathological parameters available at the time of the initial biopsy. However, subset analyses revealed that the prognostic impact of BCAR1 expression was limited to ERG-negative cancer. That BCAR1 up regulation was linked to almost all analyzed deletions (*p* < 0.0001 each for PTEN, 5q, 6q deletion) may suggest a functional link to genomic instability.

**Conclusion:**

The results of our study identify BCAR1 as a prognostic biomarker with potential clinical value for risk stratification of ERG-negative prostate cancer.

**Electronic supplementary material:**

The online version of this article (doi: 10.1186/s12885-017-3956-3) contains supplementary material, which is available to authorized users.

## Background

Prostate cancer is the most common cancer in men in Western societies [1]. At diagnosis the majority of prostate cancer is curable, but a minor subset of tumors is characterized by aggressive growth and metastasis. Despite recent advance in research for biomarkers, the established pre-treatment prognostic parameters are Gleason score, tumor extent on biopsy, pre-operative PSA and clinical parameters. These data are statistically powerful but not sufficient for optimal individual treatment choice. It is therefore hoped that the analysis of further biomarkers may lead to improved individual prediction of tumor aggressiveness in the future.

Breast cancer anti-estrogen resistance 1 (BCAR1/p130Cas) is a scaffold protein that serves as a hub in cellular signaling. It facilitates the assembly of multi-protein complexes regulating diverse cellular processes such as migration, invasion, proliferation and survival. BCAR1 participates in signal conduction of major oncogenic kinases such as Abl, FAK and Src. Consequently, BCAR1 has been shown to be overexpressed in diverse malignancies, including cancers of the breast, lung, liver and brain, and has been linked to adverse features in these entities (reviewed in [2, 3]). Initial evidence also suggests a role for BCAR1 in prostate cancer progression, as its overexpression was linked to an unfavorable tumor phenotype and biochemical relapse in three studies analyzing 110 [4], 130 [5] and 242 [6] prostate cancer specimens.

Based on these data, we intended to confirm the biologic and prognostic role of BCAR1 protein in a very large cohort of prostate cancer patients. For this purpose, we chose our tissue microarray (TMA) comprising >11,000 prostate cancer specimens with attached clinical and molecular data. Our study highlight that BCAR1 expression is associated with unfavorable tumor features and that the prognostic impact of BCAR1 is limited to ERG-negative cancers.

## Methods

### Patients

Radical prostatectomy samples were taken from 11,152 patients, undergoing surgery between 1992 and 2011 at the Department of Urology and the Martini Clinics at the University Medical Center Hamburg-Eppendorf. Follow-up was available from 9695 patients (median 36.8 months; range 1 to 228 months; Additional file 1: Table S1). Prostate specific antigen (PSA) recurrence was defined as a postoperative PSA of ≥0,2 ng/ml. Histological analysis of prostate specimen was done as detailed in [7] and TMA were produced as described earlier in [8]. Each TMA block contained various control tissues, including normal prostate tissue. The molecular database attached to this TMA contained results on Ki67 expression in 7010 (expanded from [9]), ERG expression in 9628, ERG break apart fluorescence in-situ hybridization (FISH) analysis in 6106 (expanded from [10]), and deletion status of 5q21 in 3037 (expanded from [11]), 6q15 in 3528 (expanded from [12]), PTEN in 6130 (expanded from [13]), and 3p13 in 1290 (expanded from [14]) tumors.

### Immunohistochemistry

Freshly cut TMA sections were stained on 1 day and in one experiment. Slides were deparaffinized and exposed to heat-induced antigen retrieval at 121 °C in Tris-EDTA-citrate buffer (pH 7.8). BCAR1 specific mouse monoclonal antibody (clone M144, Abcam, Cambridge, UK) was applied at 1/37.5 dilution at 37 °C for 60 min. BCAR1 staining was visualized with the EnVision Kit (Dako, Glostrup, Denmark) according to the manufacturer’s directions. Staining was localized to the cytoplasm. It was homogenous in the analyzed tissue samples and therefore staining intensity was semi quantitatively assessed as negative, weak, moderate, and strong.

### Statistics

JPM 9 software (SAS Institute Inc., NC, USA) was used. Contingency tables and the likelihood-ratio chi2-test were performed to find associations between molecular parameters and clinical tumor characteristics. Kaplan-Meier survival curves were calculated and the Log-Rank test was applied to detect differences. Cox proportional hazards regression was performed to look for statistical independence of pathological, molecular and clinical variables. Separate analyses were done using various sets of parameters available either before or after prostatectomy.

## Results

### Technical issues

Eighty four point nine percent of the 11,152 arrayed tumor samples were interpretable in our TMA analysis. 15.1% were non-informative, which included 1679 spots with lack of tissue samples or absence of unequivocal cancer tissue in the TMA spot.

### BCAR1 expression

**Fig. 1:**
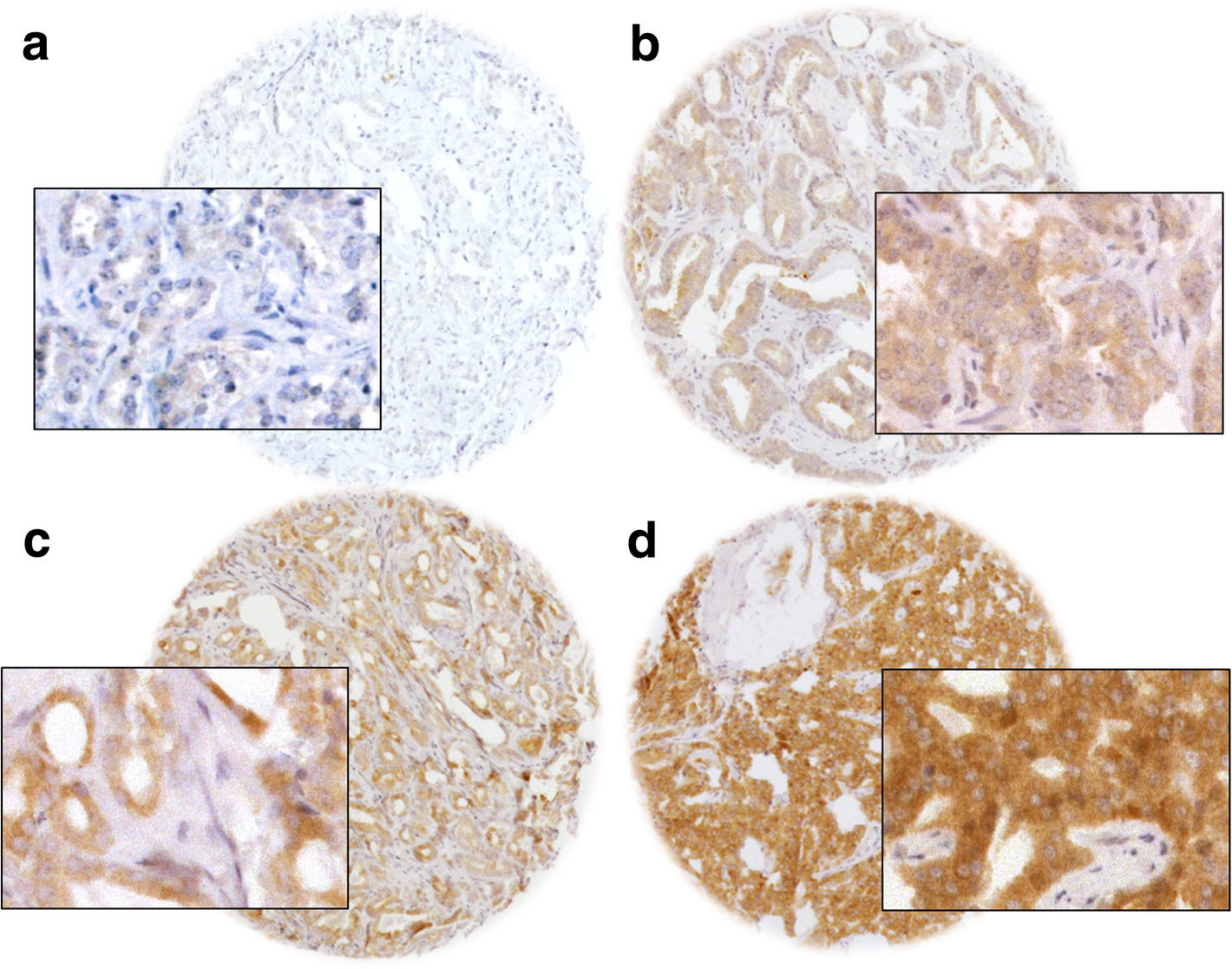
Representative image of BCAR1 expression (**a**) negative, (**b**) weak, (**c**) moderate, (**d**) strong staining at 100×, and 400× (inset) magnification

BCAR1 staining was generally absent or very faint in normal prostatic secretory cells, basal cells and stromal cells. In cancer cells, cytoplasmic BCAR1 expression was observed in 77.6% of 9472 interpretable prostate cancers; weak in 15.9%, moderate in 23.2%, and strong in 38.5% of cases. Fig. 1 shows representative images. BCAR1 expression correlated with the expression of the androgen receptor (Additional file 1: Figure S3).

### Associations with TMPRSS2:ERG fusion status and ERG protein expression

**Fig. 2:**
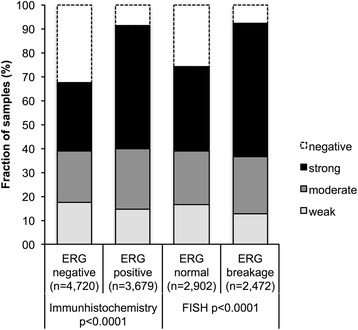
Association between BCAR1 staining and ERG-status

To evaluate whether BCAR1 expression is associated with ERG rearrangements in prostate cancers, we used pre-existing data on ERG status obtained by FISH in 5379 cancers and by IHC in 8421 tumors for which BCAR1 staining was also available. Data on both ERG FISH and ERG IHC were available from 5938 cancers, and an identical result (ERG IHC positive and rearrangement by FISH or ERG IHC negative and missing rearrangement by FISH) was found in 5666 of 5938 (95.4%) cancers. The level of BCAR1 staining was associated with the presence of ERG rearrangements and ERG expression in prostate cancers (*p* < 0.0001 each; Fig. 2). For example, moderate or strong BCAR1 staining was observed in 79.3% of cancers with ERG rearrangement detected by FISH but found in only 57.3% of cancers without such rearrangements (*p* < 0.0001).

### Associations with clinical tumor characteristics

High BCAR1 staining was associated with advanced tumor stage (*p* = 0.008), high Gleason score (*p* < 0.0001) and low preoperative PSA level (*p* < 0.0001) when all cancers were jointly analyzed (Table 1, Additional file 1: Figure S4 and S5). Subset analysis of ERG-negative and ERG-positive cancer revealed that these associations were largely driven by the subset of ERG-negative cancers (Additional file 1: Table S2), while most differences were only small in ERG-positive cancers (Additional file 1: Table S2). For example, in ERG-negative cancer, strong BCAR1 expression was found in 27.9% of pT2 cancers and increased by 9.3% to 37.2% in tumors ≥ pT3b, while the difference in ERG-positive cancer was only 1.7% between pT2 (51.9%) and ≥ pT3b (50.2%). That significant *p*-values were obtained despite these small differences is most likely due to the very high numbers of cancers included in our analysis.

**Table 1:** Association between BCAR1 staining and prostate cancer clinical characteristics

Parameter	BCAR1 (%)					
	N evaluable	Negative	Weak	Moderate	Strong	*P* value
All cancers	9472	22.4	15.9	23.2	38.5	
Tumor stage						
pT2	6170	23.7	15.9	23.1	37.4	*0.0082*
pT3a	2138	20.4	16.1	24.2	39.3	
pT3b-pT4	1160	19.5	15.6	21.7	43.2	
Gleason score						
≤ 3 + 3	2186	27.2	19.0	23.1	30.8	*< 0.0001*
3 + 4	5133	21.9	14.9	23.5	39.8	
3 + 4 Tertiary 5	345	22.9	14.5	23.8	38.8	
4 + 3	893	18.4	17.6	21.7	42.3	
4 + 3 Tertiary 5	464	15.1	14.4	22.4	48.1	
≥ 4 + 4	445	20.9	11.7	23.2	44.3	
Lymph node metastasis						
N0	5262	20.9	16.1	23.2	40.0	*<0.0001*
N+	494	21.3	12.8	21.5	44.5	
Preoperative PSA level (ng/ml)						
< 4	1480	16.9	15.0	24.0	44.1	*<0.0001*
4–10	5475	21.6	15.9	23.5	39.0	
10–20	1817	26.0	15.9	22.5	35.6	
> 20	638	32.1	17.6	19.0	31.4	
Surgical margin						
Negative	7566	22.1	15.8	23.3	38.8	*<0.0001*
Positive	1794	23.4	16.2	22.9	37.6	

### Association to cell proliferation

Increased BCAR1 staining was linked to accelerated cell proliferation. Although this association was found when all cancers were jointly analyzed (*p* < 0.0001; Table 2), subset analysis revealed that it was largely driven by ERG-negative tumors (Additional file 1: Table S3). Here, the Ki67 labeling index (Li) ranged from 1.5% (in BCAR1 negative cancer) to 4.0% (in strongly BCAR1 positive cancer, *p* < 0.0001), while this association was weaker (2.2% in BCAR1 negative vs. 3.2% in BCAR1 strong, *p* < 0.0001) in ERG-positive cancer. Further subset analysis showed that the association was independent from the Gleason score in ERG-negative cancer, because they held true in subsets of cancers with identical Gleason score. Such an unequivocal independent association was lacking in ERG-positive cancer.

**Table 2:** Association between BCAR1 expression and Ki67-labeling index in different Gleason scores

Gleason score	BCAR1 expression	Ki67-	labeling	index	*P* value
		N	Mean	± SD	
All	Negative	1422	1.6	0.07	*<0.0001*
	Weak	1063	2.5	0.08	
	Moderate	1467	2.8	0.07	
	Strong	2390	3.5	0.05	
≤3 + 3	Negative	391	1.3	0.10	*<0.0001*
	Weak	267	2.3	0.13	
	Moderate	295	2.4	0.12	
	Strong	406	2.8	0.10	
3 + 4	Negative	747	1.5	0.08	*<0.0001*
	Weak	552	2.4	0.10	
	Moderate	846	2.6	0.08	
	Strong	1387	3.3	0.06	
3 + 4 Tertiary 5	Negative	61	2.0	0.32	*<0.0001*
	Weak	45	2.6	0.37	
	Moderate	59	3.4	0.32	
	Strong	96	3.9	0.25	
4 + 3	Negative	115	1.8	0.29	*<0.0001*
	Weak	115	2.8	0.29	
	Moderate	126	3.0	0.27	
	Strong	233	4.2	0.20	
4 + 3 Tertiary 5	Negative	50	2.0	0.53	*<0.0001*
	Weak	47	3.4	0.54	
	Moderate	72	3.8	0.44	
	Strong	147	4.8	0.31	
≥4 + 4	Negative	58	3.5	0.63	*<0.0001*
	Weak	36	4.0	0.80	
	Moderate	68	4.6	0.58	
	Strong	118	5.5	0.44	

### Relationship with key genomic deletions in ERG-positive and ERG-negative prostate cancers

**Fig. 3:**
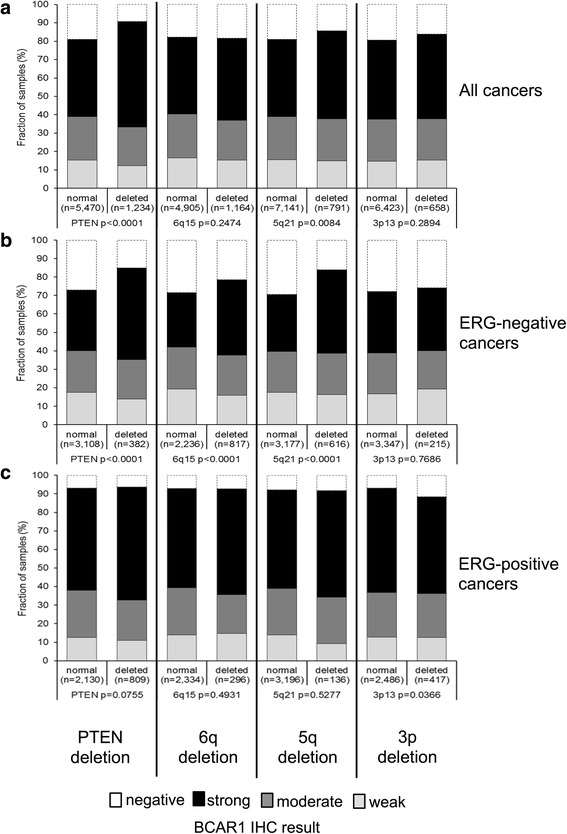
Association between BCAR1 staining and 10q23 *(PTEN*), 6q21 (*MAP3K7*), 5q21 (*CHD1*), 3p13 *(FOXP1*) deletion in (**a**) all cancers, (**b**) the ERG-negative and (**c**) -positive subset

Previous observations had prostate cancer divided in distinct molecular subgroups defined by TMPRSS2:ERG fusion and various genomic deletions. Others and us reported strong association between deletions involving the chromosomal region of PTEN and 3p13 with the presence of ERG fusions and deletions of 5q21 and 6q15 with lack of ERG fusions [11–16]. High BCAR1 staining was associated with PTEN deletion (*p* < 0.0001) and marginally associated with deletions of CHD1 (5q21) (*p* = 0.0084) when all cancers were jointly analyzed (Fig. 3a). In ERG-negative cancer, high levels of BCAR1 expression were significantly linked to presence of deletions of PTEN (*p* < 0.0001), CHD1 (5q21) (*p* < 0.0001) and MAP3K7 (6q15) (*p* < 0.0001; Fig. 3b), while these associations were lost in ERG-positive cancer (Fig. 3c).

### BCAR1 and clinical outcome

**Fig. 4:**
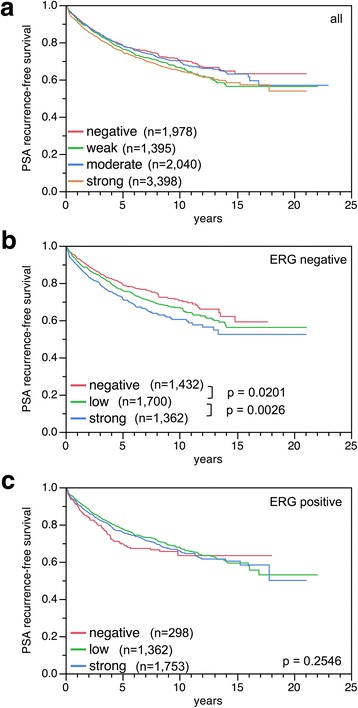
Kaplan-Meier plots of prostate specific antigen (PSA) recurrence after radical prostatectomy and BCAR1 staining in (**a**) all cancers, (**b**) the ERG-negative and (**c**) -positive subset. Low level combines cases with weak and moderate BCAR1 expression, which had similar prognosis

Follow-up was available for 8255 patients with informative BCAR1 data. Strong versus negative BCAR1 expression was associated with early PSA recurrence in all tumors (Fig. 4a) and was limited in subgroup analyses to the subset of ERG-negative cancer (Fig. 4b,c). To better understand the prognostic power of BCAR1, we performed further subset analysis in cancers with identical classical and quantitative Gleason score in all patients (Additional file 1: Figure S1) and the ERG negative subset (Additional file 1: Figure S2). Here, BCAR1 staining did not provide significant prognostic information beyond the classical Gleason score (Additional file 1: Figures S1a and S2a) or the quantitative Gleason score (Additional file 1: Figures S1b-h and S2b-h).

### Multivariate analysis

In order to test whether the prognostic impact of BCAR1 was independent from established prognostic parameters, four multivariate analyses were done to evaluate the relevance of BCAR1 expression in different clinical scenarios (Table 3). In scenario 1, the preoperatively available parameters (preoperative PSA value, clinical stage, and Gleason score at biopsy) were jointly analyzed with the BCAR1 expression level. In scenario 2, the biopsy Gleason was replaced by the Gleason score obtained at radical prostatectomy. In scenario 3 clinical stage is superseded by pathological tumor stage and surgical margin and in scenario 4 the lymph node status is added. The BCAR1 expression level remained marginally significant in pre – and postoperative scenarios with hazard ratios for PSA recurrence-free survival from 1.14 to 1.29.

**Table 3:** Hazard ratio of PSA recurrence-free survival of established prognostic parameters and BCAR1 expression

	Scenario	1	2	3	4
	Analyzable (N)	8522	8662	8778	5362
Preoperative	Gleason score biopsy				
	3 + 4 vs. ≤3 + 3	1.98 ^***^			
	4 + 3 vs. 3 + 4	1.62 ^***^			
	≥ 4 + 4 vs. 4 + 3	1.30 ^***^			
	Clinical tumor (cT) stage				
	T2a vs. T1c	1.33 ^***^	1.33 ^***^		
	T2b vs. T2a	1.47 ^***^	1.40 ^***^		
	T3a vs. T2c	0.62 ^*^	0.65 ^*^		
	Preoperative PSA level				
	4–10 vs. <4	1.45 ^***^	1.38 ^**^	1.21 ^*^	1.17
	10–20 vs. 4–10	1.52 ^***^	1.43 ^***^	1.28 ^***^	1.19 ^*^
	> 20 vs. 10–20	1.69 ***	1.53 ***	1.20 *	1.19 ^*^
	BCAR1 expression				
	Weak vs. negative	1.17 ^*^	1.14	1.16 ^*^	1.29 ^*^
	Moderate vs. weak	0.91	0.92	0.88	0.78 *
	Strong vs. moderate	1.14 ^*^	1.08	1.12	1.13
Postoperative	Gleason score prostatectomy				
	3 + 4 vs. ≤3 + 3		3.05 ^***^	2.48 ^***^	2.24 ^***^
	4 + 3 vs. 3 + 4		2.56 ^***^	2.13 ^***^	2.01 ^***^
	≥ 4 + 4 vs. 4 + 3		1.70 ^***^	1.20 ^*^	1.11
	Pathological tumor (pT) stage				
	T3a vs. T2			2.00 ^***^	2.03 ^***^
	T3b vs. T3a			1.72 ^***^	1.55 ^***^
	T4 vs. T3b			1.38 ^*^	1.35
	Surgical margin (R) status				
	R1 vs. R0			1.44 ^***^	1.31 ^***^
	Lymph node (N) status				
	N+ vs. N0				1.44 ^***^

Scenario 1 combines preoperatively available parameter (preoperative Gleason score obtained on the original biopsy, clinical tumor (cT) stage, and preoperative PSA) with the postoperative BCAR1 expression. In scenario 2 the biopsy Gleason is replaced by the Gleason score obtained on radical prostatectomy (RPE). In scenario 3 cT-stage is superseded by pathological tumor (pT) stage and surgical margin (R) status. In scenario 4 the lymph node (pN) stage is added. Asterisk indicate significance level: * *p* ≤ 0.05, ** *p* ≤ 0.001, and *** *p* ≤ 0.0001

## Discussion

The results of this study suggest that high BCAR1 expression is a weak independent predictor of unfavorable tumor characteristics and early PSA recurrence. Consistent with earlier findings [5], BCAR1 staining was barely detectable in luminal cells of non-neoplastic prostatic glands but clearly up regulated in a large fraction of prostate cancers, which suggests a role for BCAR1 during prostate cancer development. The analysis revealed cytoplasmic BCAR1 staining in 76.6% of 9495 analyzable prostate cancers. These numbers fit well to previous TMA based studies, which reported up to 90% of BCAR1 positivity in sets of 110 up to 242 prostate carcinomas [4–6].

BCAR1 up regulation was linked to aggressive cancer features in our study, including high Gleason score, advanced tumor stage, presence of lymph node metastases, rapid tumor cell proliferation and early biochemical recurrence, arguing for a contribution of elevated BCAR1 protein expression to prostate cancer progression. These findings are supported by the results of several earlier studies suggesting associations between BCAR1 up regulation and advanced prostate cancer features such as castration resistance, metastasis and early biochemical relapse [4–6]. A tumor promoting role of BCAR1 overexpression fits well to the known function of BCAR1, which serves as a hub for several oncogenic pathways regulating processes like cell proliferation, growth, migration, and other cancer relevant cellular functions (reviewed in [2, 3]).

The molecular database attached to our TMA allowed us to draw conclusions on molecular mechanisms associated with BCAR1 up regulation. It is well known that about the half of prostate cancers carry a gene fusion, which links the androgen-regulated serine protease TMPRSS2 with the ETS-transcription factor ERG resulting in an androgen-related expression of ERG with subsequent dysregulation of more than 1600 ERG target genes [17–19]. BCAR1 up regulation was strongly linked to TMPRSS2:ERG fusions in our study. ERG does not seem to be implicated in transcriptional control of BCAR1 based on the results of studies analyzing global transcriptional changes between ERG-negative and ERG-positive prostate cancers [15, 19–22]. It is, thus, possible that post-transcriptional modifications may account for the different BCAR1 expression levels in ERG-positive and ERG-negative cancers, including for example altered protein stability. This assumption is supported by studies demonstrating that ERG activation modulates the activity of the reversible protein ubiquitination cascade [15, 23], including the E3 ubiquitin ligase SKP2 that regulates stability of the BCAR1/p130CAS protein [24–26].

The association between BCAR1 expression and lymph node metastasis was puzzling because it was in the opposite direction between the ERG negative and positive cancers and significant in both subsets (Additional file 1: Table S2). We assume a complex situation as a result of 1) minor differences resulting in high statistical significance because of the very high sample numbers, 2) ERG driven sudden up-regulation of BCAR1 expression and 3) regression to the mean in tumor progression to nodal metastasis which means increase of BCAR1 staining in the *ERG negative* and decrease of BCAR1 staining in the *ERG positive* cancer subset.

In prostate cancer are after the TMPRSS2: ERG fusion, chromosomal deletions the most frequent type of genomic aberration. They occur at frequencies of up to 40% [15, 27] and are associated with poor prognosis [11–15]. Deletion of PTEN (20%), 6q (20%), 5q (10%) and 3p (10%) are linked to either positive ERG status (PTEN, 3p) or negative ERG status (6q, 5q). In our study, BCAR1 up regulation was associated with most of these deletions (PTEN, 5q21, and 6q15). This finding is consistent with earlier work linking altered BCAR1 activity to development of genetic instability [28]. It has been shown that BCAR1 can translocate to the nucleus under hypoxic conditions, where it specifically impairs the homologous repair (HR) protein RAD51 [28]. Several studies have demonstrated that RAD51 deficiency can induce replication defects, genetic instability and chromosomal rearrangements [29, 30].

In previous studies, we identified several proteins, which were also expressed at higher levels in ERG-positive than in ERG-negative prostate cancer. In some of these, the prognostic effect was likewise restricted to the ERG-negative subset [31–33]. Here we identify BCAR1 as a protein following this pattern. In opposite some other biomarker were only prognostic in ERG-positive cancer [32, 34]. Together, these data show that tumor relevant functions of BCAR1 and other proteins turn out to be attenuated or amplified by ERG. ERG seems to be a critical modifier of the intracellular environment [19, 20, 23]. These challenge the concept of a unique prognostic molecular test applicable to all patients [35, 36]. It appears possible that different tests need to be developed for ERG-positive and ERG-negative cancer. Furthermore, the small difference of about 10% in Kaplan-Meier plots between negative and strongly positive BCAR1 expression, shows that BCAR1 seems to be a weak prognostic marker (Fig. 4). Thus the BCAR1 biomarker may best aid in decision making if combined with other marker in ERG-negative prostate cancer.

It is of note that the Gleason score had the highest hazard ratio for PSA recurrence-free survival in multivariate analysis and is therefore the strongest (and less expensive) prognostic marker in prostate cancer. We demonstrated recently, that with the percentage of unfavorable Gleason patterns, Gleason grading could be transformed from a categorical into a continuous variable (i.e., the quantitative Gleason score) with subtler distinction of prognosis [37]. The power of morphological methods competing with biomarkers for predicting prostate cancer aggressiveness is best demonstrated by the separate analysis of different prognostic Gleason groups. That the prognostic impact of BCAR1 was lost in groups defined by classical Gleason score categories or by the quantitative Gleason score demonstrates how difficult it is for a biomarker to outperform a morphological malignancy score.

## Conclusions

The results of our study demonstrate that BCAR1 is an ERG subset specific prognostic biomarker. The minor prognostic difference between cancers with negative or strong BCAR1 expression limits its clinical impact as a stand-alone marker. However, BCAR1 may be a useful marker if combined with other molecular markers, especially for ERG-negative prostate cancer.

## Additional files

Additional file 1: **Figure S1.** Kaplan-Meier plots of prostate specific antigen (PSA) recurrence after radical prostatectomy and negative or strong BCAR1 staining in subsets of *all* cancers defined by (a) classical Gleason score, (b-h) quantitative Gleason score defined by the percentage of Gleason 4 grade and (i-j) by the tertiary Gleason 5 grade. **Figure S2.** Kaplan-Meier plots of prostate specific antigen (PSA) recurrence after radical prostatectomy and BCAR1 staining in subsets of *ERG negative* cancers defined by (a) classical and (b-h) quantitative Gleason score, defined by the percentage of Gleason 4 grade and (i-j) by the tertiary Gleason 5 grade. **Figure S3.** Correlation of BCAR1 staining and androgen receptor (AR) staining in *all* cancers, **Figure S4.** Kaplan-Meier plot of prostate specific antigen (PSA) recurrence after radical prostatectomy and clinical stage in *all* cancers, **Figure S5.** Kaplan-Meier plot of prostate specific antigen (PSA) recurrence after radical prostatectomy and Gleason score at biopsy in *all* cancers, **Table S1.** Pathological and clinical data of the arrayed prostate cancer, **Table S2.** Association between BCAR1 staining and prostate cancer clinical characteristics in ERG–fusion negative and positive subsets, **Table S3.** Association between BCRA1 expression and Ki67-labeling index depending on ERG-fusion status in different Gleason scores. (DOC 5580 kb)

## Abbreviations

BCAR1: Breast cancer anti-estrogen resistance 1
CHD1: Chromodomain-helicase-DNA-binding protein 1
ERG: Erythroblast transformation-specific (ETS) related gene
FISH: Fluorescence in situ hybridization
FOXP1: Fork head box protein P1
Ki67LI: Ki67 labeling index
MAP3K7: Mitogen-activated protein kinase kinase kinase 7
PSA: Prostate specific antigen
PTEN: Phosphatase and tensin homolog
TMA: Tissue microarray
TMPRSS2: Trans membrane protease, serine 2
