# Supplementary material for: High BCAR1 expression is associated with early PSA recurrence in ERG negative prostate cancer
Source: BMC Cancer. 2018 Jan 5;18:37. doi: 10.1186/s12885-017-3956-3 (PMC5756403; doi:10.1186/s12885-017-3956-3)
Supplement: Supplementary file 1 — Figure S1. Kaplan-Meier plots of prostate specific antigen (PSA) recurrence after radical prostatectomy and negative or strong BCAR1 staining in subsets of all cancers defined by (a) classical Gleason score, (b-h) quantitative Gleason score defined by the percentage of Gleason 4 grade and (i-j) by the tertiary Gleason 5 grade. Figure S2. Kaplan-Meier plots of prostate specific antigen (PSA) recurrence after radical prostatectomy and BCAR1 staining in subsets of ERG negative cancers defined by (a) classical and (b-h) quantitative Gleason score, defined by the percentage of Gleason 4 grade and (i-j) by the tertiary Gleason 5 grade. Figure S3. Correlation of BCAR1 staining and androgen receptor (AR) staining in all cancers, Figure S4. Kaplan-Meier plot of prostate specific antigen (PSA) recurrence after radical prostatectomy and clinical stage in all cancers, Figure S5. Kaplan-Meier plot of prostate specific antigen (PSA) recurrence after radical prostatectomy and Gleason score at biopsy in all cancers, Table S1. Pathological and clinical data of the arrayed prostate cancer, Table S2. Association between BCAR1 staining and prostate cancer clinical characteristics in ERG–fusion negative and positive subsets, Table S3. Association between BCRA1 expression and Ki67-labeling index depending on ERG-fusion status in different Gleason scores. (DOC 5580 kb) [file 12885_2017_3956_MOESM1_ESM.doc]

**Supporting Information**

**
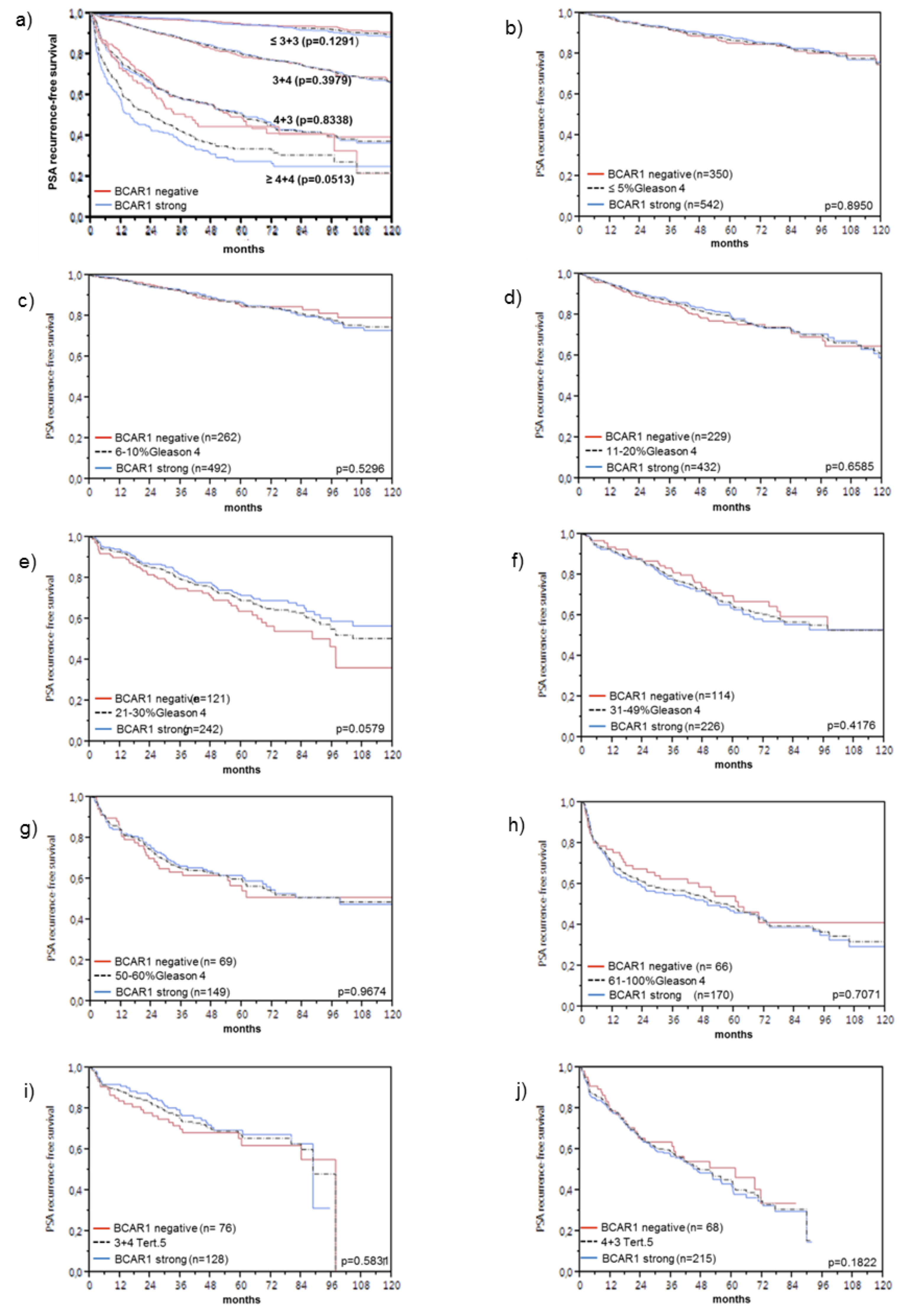
**

**Fig S1**. Kaplan-Meier plots of prostate specific antigen (PSA) recurrence after radical prostatectomy and negative or strong BCAR1 staining in subsets of *all* cancers defined by (a) classical Gleason score, (b-h) quantitative Gleason score defined by the percentage of Gleason 4 grade and (i-j) by the tertiary Gleason 5 grade.

**
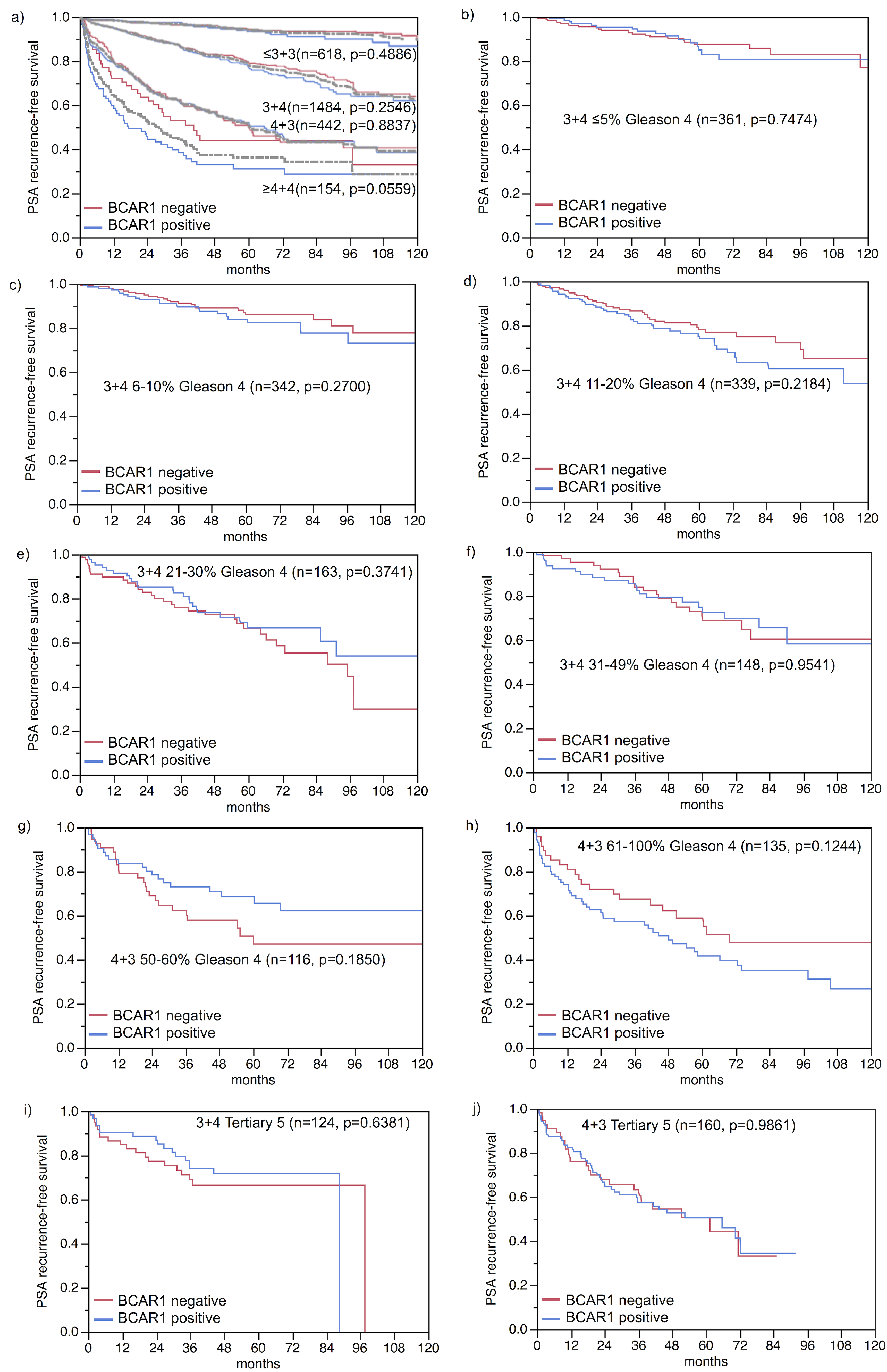
**

**Fig S2**. Kaplan-Meier plots of prostate specific antigen (PSA) recurrence after radical prostatectomy and BCAR1 staining in subsets of *ERG negative* cancers defined by (a) classical and (b-h) quantitative Gleason score, defined by the percentage of Gleason 4 grade and (i-j) by the tertiary Gleason 5 grade.

**
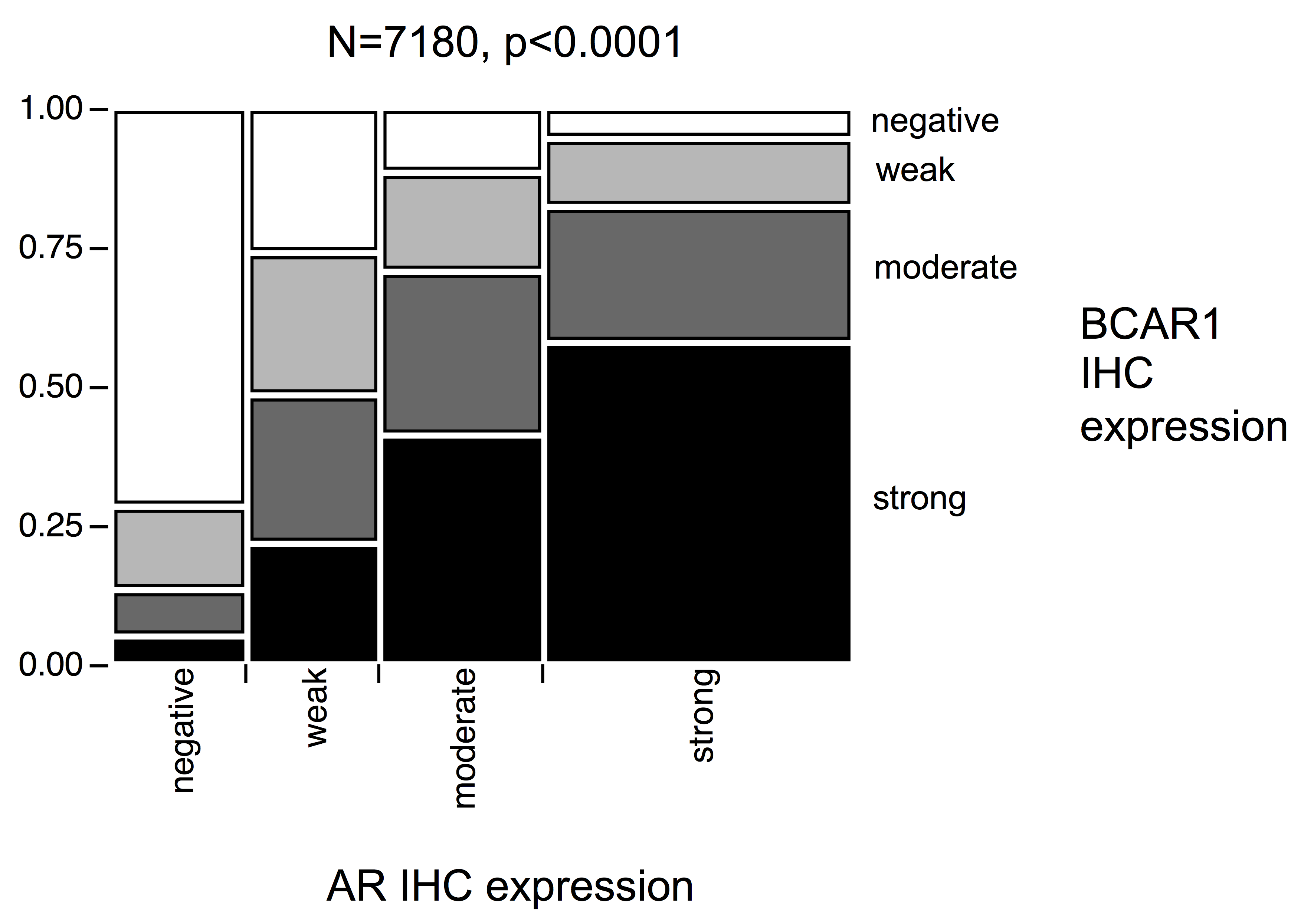
**

**Fig S3**. Correlation of BCAR1 staining and androgen receptor (AR) staining in *all* cancers

**
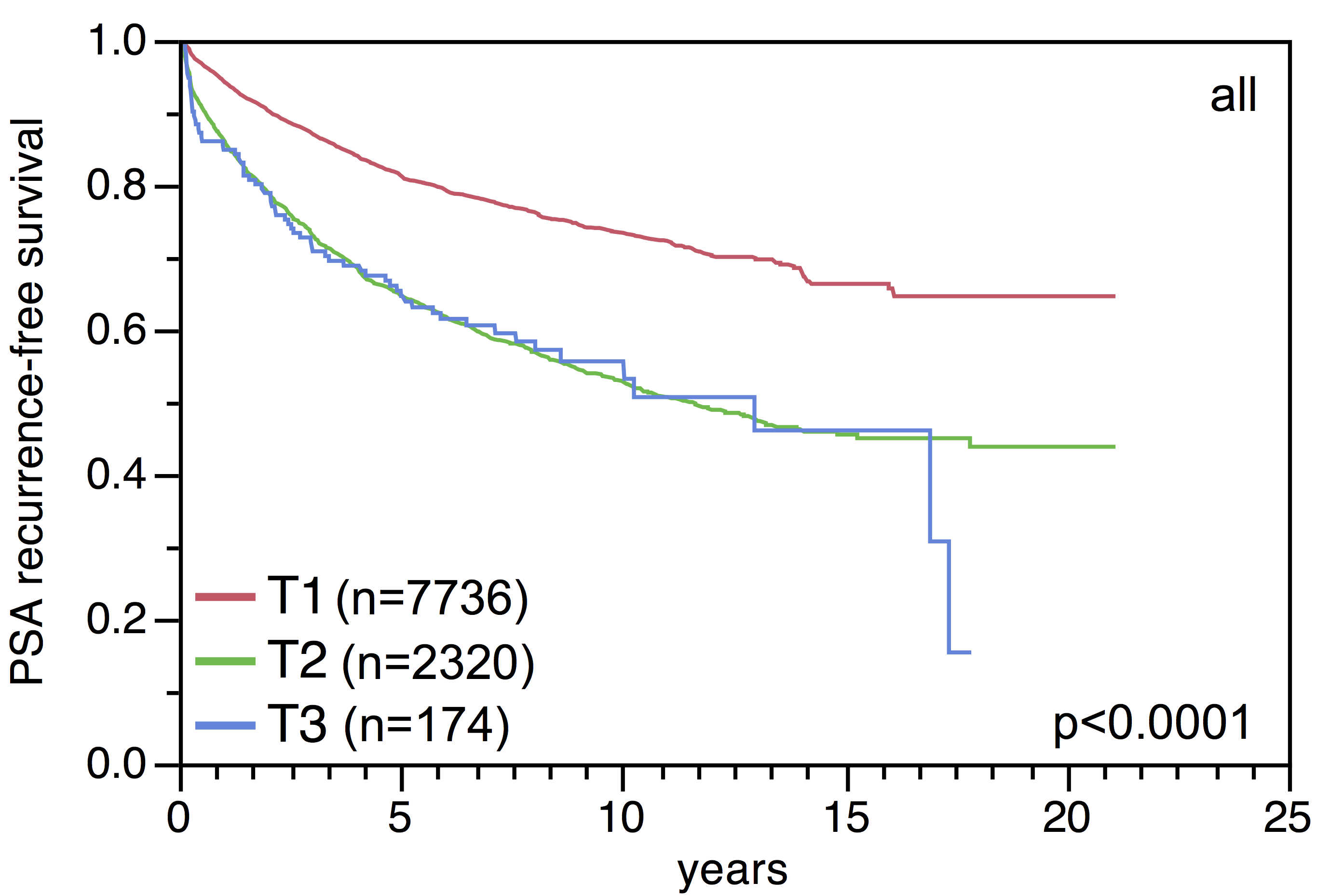
**

**Fig S4**. Kaplan-Meier plot of prostate specific antigen (PSA) recurrence after radical prostatectomy and clinical stage in *all* cancers

**
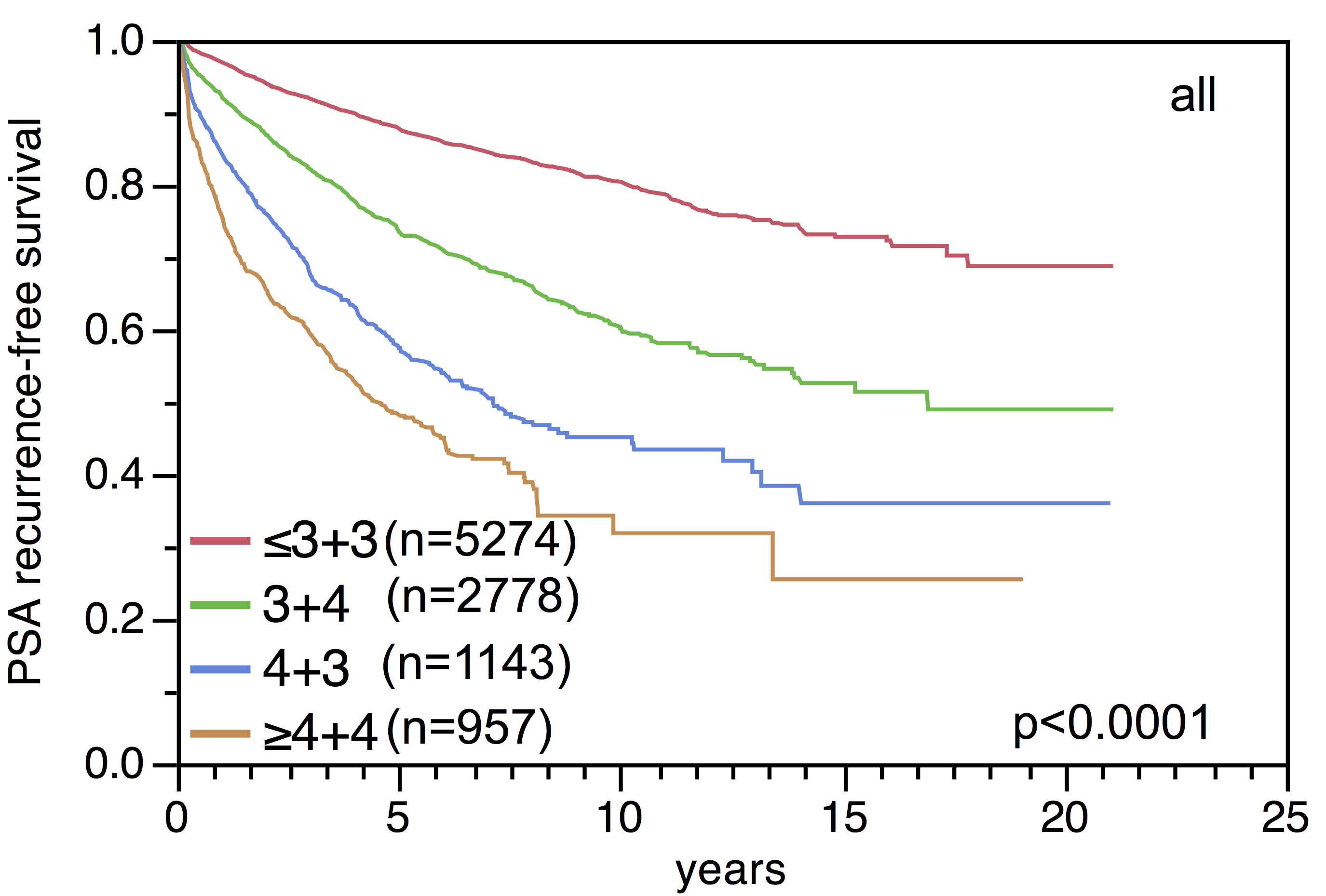
**

**Fig S5**. Kaplan-Meier plot of prostate specific antigen (PSA) recurrence after radical prostatectomy and Gleason score at biopsy in *all* cancers

**Table S1.** Pathological and clinical data of the arrayed prostate cancers

|  | **No. of patients (%)** | |
| --- | --- | --- |
|  | **Study cohort on TMA** | **Biochemical relapse** |
|  | **n=11152** | **among category** |
| **Follow-up (mo)** |  |  |
| N | 10376 | 2582 (24.9%) |
| Mean | 65.6 | - |
| Median | 60 | - |
| **Age** (y) |  |  |
| ≥50 | 321 | 58 (18.1%) |
| 51-59 | 2772 | 613 (22.1%) |
| 60-69 | 6567 | 1542 (23.5%) |
| ≥70 | 1440 | 369 (25.6%) |
| **Pretreatment PSA (ng/ml)** | |  |
| <4 | 1414 | 205 (14.5%) |
| 4-10 | 6759 | 1283 (19.0%) |
| 10-20 | 2169 | 703 (32.4%) |
| >20 | 725 | 379 (52.3%) |
| **pT category (AJCC 2002)** | |  |
| pT2 | 7371 | 914 (12.4%) |
| pT3a | 2409 | 826 (34.3%) |
| pT3b | 1262 | 793 (62.8%) |
| pT4 | 63 | 49 (77.8%) |
| **Gleason score** |  |  |
| ≤3+3 | 2735 | 230 (8.4%) |
| 3+4 | 5961 | 1167 (19.6%) |
| 3+4 Tertiary 5 | 379 | 107 (28.2%) |
| 4+3 | 1056 | 521 (49.3%) |
| 4+3 Tertiary 5 | 520 | 265 (51.0%) |
| ≥4+4 | 491 | 291 (59.3%) |
| **pN category** |  |  |
| pN0 | 6119 | 1632 (26.7%) |
| pN+ | 561 | 379 (67.6%) |
| **Surgical margin** |  |  |
| Negative | 8999 | 1680 (18.7%) |
| Positive | 2108 | 902 (42.8%) |
| NOTE: Numbers do not always add up to 11152 in the different categories because of cases with missing data. Percent in column "Biochemical relapse among category" refers to the fraction of samples with biochemical relapse within each parameter in the different categories. Abbreviation: AJCC, American Joint Committee on Cancer. | | |

**Table S2. Association between BCAR1 staining and prostate cancer characteristics in ERG–fusion negative and positive tumor**

|  | **BCAR1 IHC** | | | | | | | | | | | |
| --- | --- | --- | --- | --- | --- | --- | --- | --- | --- | --- | --- | --- |
| **Parameter** | **ERG-negative subset** | | | | | | **ERG-positive subset** | | | | | |
|  | **N** | **Negative (%)** | **Weak  (%)** | **Moderate (%)** | **Strong (%)** | **P** | **N** | **Negative (%)** | **Weak (%)** | **Moderate (%)** | **Strong (%)** | **P** |
|  |  |  |  |  |  |  |  |  |  |  |  |  |
| **All cancers** | 4719 | 32.6 | 17.3 | 21.4 | 28.7 |  | 3679 | 8.5 | 14.6 | 25.5 | 51.4 |  |
| **Tumor stage** | |  |  |  |  |  |  |  |  |  |  |  |
| pT2 | 3194 | 34.0 | 17.4 | 20.8 | 27.9 | *0.0002* | 2175 | 7.5 | 14.4 | 26.2 | 51.9 | 0.0684 |
| pT3a | 947 | 32.4 | 17.2 | 24.1 | 26.3 |  | 1020 | 8.8 | 14.7 | 25.4 | 51.1 |  |
| pT3b-pT4 | 576 | 25.2 | 17.2 | 20.5 | 37.2 |  | 482 | 12.7 | 14.5 | 22.6 | 50.2 |  |
| **Gleason score** | |  |  |  |  |  |  |  |  |  |  |  |
| ≤3+3 | 1021 | 40.6 | 19.2 | 20.0 | 20.3 | *< 0.0001* | 784 | 9.3 | 19.9 | 26.9 | 43.9 | *< 0.0001* |
| 3+4 | 2484 | 32.9 | 17.1 | 21.8 | 28.2 |  | 2128 | 8.0 | 12.7 | 25.7 | 53.6 |  |
| 3+5 Tertiary 5 | 206 | 30.1 | 18.0 | 20.4 | 31.6 |  | 114 | 9.7 | 9.7 | 31.6 | 49.1 |  |
| 4+3 | 474 | 25.3 | 17.5 | 21.5 | 35.7 |  | 347 | 8.9 | 18.2 | 21.0 | 51.9 |  |
| 4+3 Tertiary 5 | 259 | 22.0 | 16.2 | 20.1 | 41.7 |  | 172 | 5.8 | 12.2 | 25.6 | 56.4 |  |
| ≥4+4 | 273 | 24.5 | 12.5 | 24.9 | 38.1 |  | 130 | 13.9 | 10.0 | 19.2 | 56.9 |  |
| **Lymph node metastasis** | | |  |  |  |  |  |  |  |  |  |  |
| N0 | 2676 | 30.38 | 17.12 | 21.97 | 30.53 | *< 0.0001* | 2077 | 7.9 | 14.6 | 25.0 | 52.5 | *0.0015* |
| N+ | 245 | 24.49 | 12.65 | 20.82 | 42.04 |  | 208 | 17.8 | 14.4 | 21.6 | 46.2 |  |
| **Preoperative PSA level (ng/ml)** | | |  |  |  |  |  |  |  |  |  |  |
| <4 | 650 | 27.9 | 15.9 | 24.3 | 32.0 | *0.0015* | 611 | 5.9 | 12.8 | 23.4 | 57.9 | *<0.0001* |
| 4-10 | 2691 | 32.0 | 17.5 | 21.6 | 29.0 |  | 2157 | 7.7 | 14.2 | 26.0 | 52.1 |  |
| 10-20 | 996 | 35.5 | 16.3 | 20.4 | 27.8 |  | 657 | 9.7 | 16.9 | 26.2 | 47.2 |  |
| >20 | 356 | 38.8 | 20.2 | 16.9 | 24.2 |  | 227 | 18.5 | 15.4 | 23.4 | 42.7 |  |
| **Surgical margin** | | |  |  |  |  |  |  |  |  |  |  |
| Negative | 3789 | 32.1 | 17.2 | 21.9 | 28.8 | 0.5462 | 2869 | 7.9 | 14.7 | 25.1 | 52.3 | 0.0674 |
| Positive | 847 | 34.3 | 17.7 | 19.3 | 28.7 |  | 768 | 10.6 | 14.2 | 27.1 | 48.2 |  |

**Table S3. Association between BCRA1 expression and Ki67-labeling index depending on ERG-fusion status in different Gleason scores**

| **Gleason** | **BCRA1** | **ERG-negative subset** | | | | **ERG-positive subset** | | | |
| --- | --- | --- | --- | --- | --- | --- | --- | --- | --- |
| **score** | **expression** | **N** | **Ki67-LI Mean** | **± SD** | **P Value** | **N** | **Ki67-LI Mean** | **± SD** | **P Value** |
| **Total** | **Negative** | 1157 | 1.5 | 0.08 | *<0.0001* | 238 | 2.2 | 0.17 | *<0.0001* |
|  | **Weak** | 618 | 2.4 | 0.11 |  | 415 | 2.7 | 0.13 |  |
|  | **Moderate** | 714 | 2.9 | 0.10 |  | 732 | 2.7 | 0.10 |  |
|  | **Strong** | 930 | 4.0 | 0.09 |  | 1421 | 3.2 | 0.07 |  |
| **≤3+3** | **Negative** | 321 | 1.2 | 0.12 | *<0.0001* | 54 | 1.8 | 0.27 | 0.0855 |
|  | **Weak** | 132 | 2.0 | 0.18 |  | 117 | 2.6 | 0.19 |  |
|  | **Moderate** | 123 | 2.6 | 0.19 |  | 166 | 2.4 | 0.16 |  |
|  | **Strong** | 129 | 3.4 | 0.18 |  | 269 | 2.6 | 0.12 |  |
| **3+4** | **Negative** | 580 | 1.4 | 0.09 | *<0.0001* | 122 | 1.9 | 0.21 | *<0.0001* |
|  | **Weak** | 310 | 2.2 | 0.13 |  | 189 | 2.7 | 0.17 |  |
|  | **Moderate** | 386 | 2.6 | 0.11 |  | 407 | 2.7 | 0.11 |  |
|  | **Strong** | 470 | 3.5 | 0.10 |  | 856 | 3.2 | 0.08 |  |
| **3+4 Tertiary 5** | **Negative** | 54 | 2.0 | 0.33 | *0.0003* | 7 | 2.3 | 0.95 | 0.5584 |
|  | **Weak** | 35 | 2.5 | 0.42 |  | 9 | 3.4 | 0.84 |  |
|  | **Moderate** | 30 | 3.6 | 0.45 |  | 28 | 3.2 | 0.47 |  |
|  | **Strong** | 49 | 4.0 | 0.35 |  | 46 | 3.7 | 0.37 |  |
| **4+3** | **Negative** | 83 | 1.7 | 0.38 | *<0.0001* | 18 | 2.6 | 0.67 | *0.0180* |
|  | **Weak** | 55 | 3.2 | 0.47 |  | 41 | 2.5 | 0.44 |  |
|  | **Moderate** | 65 | 3.2 | 0.43 |  | 48 | 3.2 | 0.41 |  |
|  | **Strong** | 119 | 4.5 | 0.32 |  | 95 | 4.0 | 0.29 |  |
| **4+3 Tertiary 5** | **Negative** | 45 | 1.8 | 0.56 | *<0.0001* | 6 | 3.2 | 1.47 | 0.9693 |
|  | **Weak** | 33 | 3.3 | 0.65 |  | 12 | 3.9 | 1.04 |  |
|  | **Moderate** | 33 | 3.7 | 0.65 |  | 39 | 3.9 | 0.58 |  |
|  | **Strong** | 75 | 5.6 | 0.43 |  | 68 | 3.8 | 0.44 |  |
| **≥4+4** | **Negative** | 36 | 3.0 | 0.69 | *0.0113* | 9 | 6.2 | 2.12 | 0.8371 |
|  | **Weak** | 20 | 3.8 | 0.92 |  | 9 | 4.8 | 2.12 |  |
|  | **Moderate** | 42 | 5.0 | 0.64 |  | 14 | 4.0 | 1.70 |  |
|  | **Strong** | 60 | 5.8 | 0.53 |  | 41 | 5.5 | 1.00 |  |
